# Accelerating CO_2_ electrochemical conversion towards industrial implementation

**DOI:** 10.1038/s41467-023-43762-6

**Published:** 2023-12-01

**Authors:** Doris Segets, Corina Andronescu, Ulf-Peter Apfel

**Affiliations:** 1 Institute for Energy and Materials Processes—Particle Science and Technology, University of Duisburg-Essen, Carl-Benz-Str. 199, 47057 Duisburg, Germany; 2 Center for Nanointegration Duisburg-Essen (CENIDE), Carl-Benz-Str. 199, 47057 Duisburg, Germany; 3 Chemical Technology III, Faculty of Chemistry University of Duisburg-Essen, Carl-Benz-Straße 199, 47057 Duisburg, Germany; 4 Fraunhofer Institute for Environmental, Safety and Energy Technology UMSICHT, Osterfelderstraße 3, 46047 Oberhausen, Germany; 5 Inorganic Chemistry I—Technical Electrochemistry, Ruhr University Bochum, Universitätsstraße 150, 44780 Bochum, Germany

**Keywords:** Chemistry, Engineering, Energy science and technology

## Abstract

Despite significant progress in CO2 conversion field, there remains a significant gap between fundamental research and the industrial demands. This Comment discusses key performance parameters for industrial applications and outlines current limitations in the field.

## Teaser

The electrochemical conversion of carbon dioxide by means of renewable electricity holds great promise. However, despite significant progress in current literature, there remains a significant gap between fundamental research and the industrial demands to establish new disruptive technologies in real world applications. This gap primarily arises from a mismatch between performance parameters and requirements in both areas, leading to significant challenges in technology transfer. We herein suggest pathways to bridge this gap and outline current limitations in the field, proposing key parameters and procedures towards accelerated and streamlined technology development.

Anthropogenic emissions of CO_2_ make a significant contribution to global warming. To reduce CO_2_ emissions, efforts are currently undertaken to recycle the generated CO_2_ as carbon source and at the same time enable alternative pathways for the generation of basic chemicals. Especially the integration of renewable energies is suitable to transform CO_2_ into reduced carbon compounds (e.g., CO, formate, ethylene)^1^.

Gas diffusion electrodes (GDEs) operated in flow cells enable efficient mass transport of CO_2_ to achieve high current densities^2^. However, though GDEs are most suitable for industrial systems, their performance is influenced by various parameters beyond the catalyst material, such as the GDE composition, structure, and operating conditions^2–4^. Recent studies have identified best practices for improving GDE performance, including analyzing anode gas flows, optimizing volumetric flows, and tailoring the catalyst/electrode structure^5^.

Despite these advances, there still exists a major gap between industrial and academic research, hindering the ultimate global transition of the technology into applicable systems. The gap primarily arises due to differences in parameters studied in fundamental research and those needed for industrial implementation, along with a lack of communication among stakeholders.

In this comment, we emphasize key performance parameters for industrial applications. We specifically address crucial metrics for comparing various CO_2_ reduction (CO_2_R) reports and new electrode/cell designs. This comparison might enable the identification of feedback loops, facilitating the exploration of an optimal system configuration and the determination of influential parameters for industrial-scale CO_2_ reduction.

## Performance parameters for industrial applications (Table 1)

When evaluating new catalysts, their performance is typically judged by the achieved half-cell potentials at defined current densities and Faradaic efficiencies. However, trends and mechanisms observed at low current densities (<50 mA cm^−2^) may not be applicable at economically feasible current densities (>200 mA cm^−2^). Furthermore, while half-cell potentials are necessary to understand and improve upon voltage losses or selectivity changes close to the catalytic micro-environment, from a system-standpoint, the catalytic overpotential has only minimal contribution to the overall cell voltage^6^. It should likewise be noted that the performance of a new catalyst depends on its reaction environment and can be optimized by adjusting the electrode architecture and surrounding electrolyte environment. Thus, the overpotential should not be the only criterion for judging the performance of a GDE. We therefore suggest to provide both half-cell and full cell data in future reports.

**Table 1:** Minimum requirements for industrial applications and comparison to the current state-of-the-art for a spectrum of CO_2_ reduction products (CO, HCOOH & C_2_H_4_)

Entry	Key performance parameters	Current state-of-the-art
Current density	>0.2 A cm^−2^ ^32^	0.5–1.8 A cm^−2^ ^32–34^
Energy efficiency	>50%^32^	ca. 40%^35^
Faradaic efficiency	>80%^32^	80–90%
Full cell potential @ 300 mA cm^−2^	<3.0 V, better <2.5 V, ideally <2.0 V^10,32,36^	2.7 V^37^
Single pass conversion	>50%^9^	30–70%^33,35,37^
System temperature	60–90 °C^34^	40–80 °C^37–39^
**Additional Notes & Challenges**		
Stability	<10 µV h^−1^ ^7,38^	
Cost of electricity	<0.04 USD kW h^−1^ ^9,40^	
Carbon balance	Use of internal standards – Quantification of the anode gas stream^5^	
Membranes of choice	AEMs – more energy efficient BPMs are required^36,41^	
Purity of substrates	Minimum vol % of CO_2_ in gas-stream – ca. 10%^42^	Minimization of NO_x_ and O_2_ impurities^21,43^

While the Key Performance Indicators (KPIs) are partially already achieved, particularly the cost of the electrolyzer and the stability require improvement^a^.

^a^Notably while some of the reported values could possibly not mirror directly achievable KPIs in an industrial scale, they still offer important perspectives on possibly achievable values for CO_2_ electrolysis in the future.

Instead of just presenting the cell voltage after stabilization, our suggestion is to include multiple potential curves, each with error ranges, to thoroughly evaluate the system stability. Additionally, long-term studies are crucial for assessing electrode/cell stability. Since CO_2_ electrolyzers are expected to perform at time-scales similar to H_2_-producing electrolyzers of >50.000 h, similar decay values could be employed. Overall, we should test our systems/catalysts for the maximal possible duration. Yet, long-term stability testing should focus on the highest achieved current density in a report. Furthermore, industrial systems operate at higher temperatures than typical lab-scale tests, e.g., to minimize cooling costs. Thus, developed catalysts, electrodes, and membranes are suggested to undergo stress-tests between 60 and 90 °C to understand their application potential. On a side note, we believe that herein, a commercial opportunity is also presented to system developers towards the development of tailored CO_2_-testing setups for long-term investigations, as in the case of fuel cells and hydrogen electrolyzers test stands.

Especially, focusing on the three major CO_2_R products with the highest promise towards direct industrial applicability, namely CO, HCOOH, and C_2_H_4_, the Faraday efficiency for a specific product should be preferably as high as possible (>80%) and stable over extended periods (ΔFE/Δ*t*:<0.1% per 1000 h^−1^) with minimal voltage decay rates (<10 µV/h) to ensure system stability^7^. We implore the academic community to consider what a stability test might look like, one capable of unraveling both early stages and nuances of degradation while enabling extrapolation. On the other hand, for products such as alcohols and acids, deviations from our proposed values could be considered, since such product groups usually have a higher market value^8^. Nevertheless, we believe that catalysts/electrolyzers should be optimized for the generation of one liquid product. Complex liquid mixtures containing both acids and alcohols will eventually only elevate the downstream separation costs. While it is possible to determine a target voltage decay rate based on the desired run-time and cut-off voltage increase for the electrolyzer, the same does not apply to the target cell voltage. Different techno-economic studies cite varying target values for cell voltage, depending on the specific CO_2_ electrolyzer used and considering downstream, CO_2_ capture, and regeneration costs. Overall, multiple studies converge on some ideal ranges. A cell voltage of <3.0 V at 300 mA cm^2^ is suggested to ensure industrial applicability, with values below 2.5 V and 2.0 V (after long-term operation) considered even more attractive^8–10^. Evidently, such metrics can only become clearer as electrolyzers become tested at pilot or demonstration scale to accommodate real-life data for techno-economics.

## Coherent workflows for navigating the parameter space

A main challenge in electrocatalysis is the apparent lack of reproducibility due to testing in different cell configurations and hidden parameters that are overlooked during materials and GDE development. These concealed parameters encompass factors such as whether the reaction halts during sampling or if rinsing steps are incorporated, whether operando sampling is conducted, the employment of an inert internal standard in CO_2_ gas streams for gas analysis, and the quantification of CO_2_ in both anode and cathode gas streams to ascertain product compositions and gain insights into crossover effects. Additionally, information on electrode properties and structure including e.g., adhesion, porosity, and hydrophobicity should be provided. Therefore, we propose the development of coherent workflows and protocols for each electrocatalyst, bridging synthesis, electrode and GDE fabrication, and testing (including details about the used electrolysis cells) as is common for batteries and photovoltaics and as recently was suggested for the electrochemical N_2_ reduction^11^. All information, including unexpected, apparently “negative results” like structural aging and dissolution, should be reported until all hidden parameters are deciphered and the design chain is understood. Parameters that need to be clearly described to allow any reproducibility of the system are given, as a starting point, in Table 2.

**Table 2:** Important parameters that should be reported to ensure reproducibility

Catalysts	Electrodes	Electrolysis Cells
Composition of the material (also including impurities)	Composition of the electrode	Compression of the cell
Particle size distribution, polarity	Detailed assembly method incl. ink recipe and preparation	Operation mode of the cell (single pass, semi batch, batch)
Materials morphology, aggregation state	In-plane and through-plane electrical conductivity of the electrode	Membrane used and its pretreatment
Post-mortem analysis of the catalysts via spectroscopy, diffractometry and microscopy methods as well as operando studies to observe any catalyst changes	Data sheet of manufacturer of materials used— link to product or product numbers	Thickness of each individual component (e.g., porous transport layer)
[Approximate cost for 1 g, 1 kg of electrocatalyst]	Porosity, adhesion, surface roughness and lateral homogeneity	Periphery applied (e.g., humidification & purification systems, ion exchange systems used for water purification)
	Water sorption and contact angle	Temperature inside and outside of the cell
	Electrode thickness	Geometry of flow fields used
	Active area of used electrodes—geometric flow rate in respect to active area, reporting on the occurrence of delamination	Measurement protocol
		ICP-OES analysis of the used electrolyte and anolyte
		Schematics of used cells

## Single-pass conversion

In addition, single-pass conversion uniquely informs us about an individual system’s efficiency and serves as a key metric for assessing an electrocatalyst’s true selectivity at varying current densities, even when the molar excess of CO_2_ is minimal. While theoretically, the CO_2_R could reach single-pass conversion (SPC) values up to 100%, meaning that all CO_2_ that flows into the cell is converted into any product—the achievable real-life values are based on the reactor architecture and scale. While AEM-based electrolyzers are limited to a SPC value of 50 % for C_1_ products, such as CO, and 25% for ethylene respectively due to carbonate formation, bipolar membranes (BPMs) and acidic electrolytes have shown SPC values above 70%^9,12^. In addition, depletion of CO_2_ across the length of large-scale flow-fields (>100 cm^2^) becomes an additional hurdle that researchers must take into account when developing industrially relevant systems^13,14^. Nevertheless, achieving elevated SPC values should not be performed at any cost as the maximization of cost-benefits is a fine balance of all operational parameters. Therefore, reporting of the outlet composition and overall produced amount of CO_2_R products is paramount when focusing on industrial application^15^. Alongside the electricity costs, at the industrial scale, the outlet composition is not only important for downstream separation and processing of the products, but also during the viability assessment stage of a new catalyst/electrolyzer design and scale-up^16,17^. Accordingly, we believe that downstream processes and direct utilization of electrolyzer outstreams will be a relevant and interesting topic for our community. Firstly, efficiently manufacturing and enhancing the stability and energy efficiency of model electrolyzers/stacks with active areas >100 cm^2^ under application conditions is important. Secondly, in the laboratory scale, focusing on the development of catalytic layers and electrodes capable of efficient operation with minimal excesses of CO_2_ is necessary^18^. In this context, reporting the *λ* value, which describes the stochiometric excess compared to the theoretical conversion of CO_2_ is recommended^19^.

## Separation

CO_2_R generates multiple products, necessitating the separation of resulting mixtures. Downstream of every CO_2_ electrolysis stack follows a complex system of separation and purification units^16,20^. Reaching not only highly concentrated product mixtures but also realistically separable ones will significantly facilitate the adoption of CO_2_ electrolysis by industry.

## CO_2_ source

While most research utilizes ultra-pure gases, and some reports investigated the effect of dilute CO_2_ feeds, industrial CO_2_ streams currently impose unaddressed challenges such as mixtures of trace elements that can alter the observed CO_2_ conversion activity or poison the catalysts^21^. This is not regularly considered but important to establish robust electrochemical systems with required >50.000 working hours of industrial plants. When new materials are reported, to open the path to industrial relevance, such claims must be backed up with proper stability measurements for real-life scenarios, including gas mixtures^22^. Overall, we suggest to focus on the application of promising CO_2_R approaches close to unavoidable CO_2_ sources, such as cement plants, which require the continuous conversion of thousands of tons of CO_2_ per year. Smaller specialized units, especially those coupled with bio-reactors, could pave the way for small-scale applications of CO_2_ electrolysis in a decentralized manner in the future. However, such assessments must be conducted on a case-by-case basis to better customize the specific CO_2_ electrolytic unit for the respective application.

## Setup and benchmarking

In CO_2_ conversion research, it’s widely agreed that integrating electrocatalysts into optimal electrode structures is crucial. Results using Gas Diffusion Electrodes (GDEs) reflect the entire system, including the reaction setup, to unlock its full potential from catalyst to industrial application—an essential consideration^23^.

Although commercial cell solutions exist, few reports have investigated how varying testing setups affect stability and performance. This knowledge gap has also led to a lack of experimental protocols towards set-up optimization, such as cell compression, flow field structure, water content determination and management. This is crucial since even small changes in the setup can lead to significant alterations in overall performance, as demonstrated by fuel cells and electrolyzers. Therefore, when moving to higher technology readiness levels (TRL) we clearly envision opening new research and collaboration opportunities between academia and industry, beyond the development of catalysts and GDEs^24,25^.

It is important to also emphasize the interplay between the anode and cathode in the operation of cells using membrane electrode assemblies (MEAs). The chemical reactions occurring at the anode and cathode directly impact each other. Therefore, when comparing results to existing literature, it is essential to have a reference for the specific MEA assembly used, as without it, the comparison of cathode and anode materials may not be valid^26^. Yet, this interaction does not display the only reason for a need for a proper benchmark. Likewise, altered cell resistances or mass transport in the system can lead to changes in performance. Even an altered measurement protocol can in the end, lead to different data sets^27^.

Distinguishing between the impact of catalyst, electrode, and cell characteristics becomes thus an immense challenge. We believe that it is crucial for the community to adopt catalytic benchmarks and standardized measurement protocols, as well as focus on the development of CO_2_R-tailored accelerated stress protocols. This will clearly elucidate each investigation’s contribution to the state-of-the-art, enabling all labs to validate setup efficiency and facilitate truthful comparisons between various MEA configurations.

This benchmark should be frequently used, and the resulting data should be made available according to the FAIR principles, becoming a clear repository of the influence of cell parameters and electrode adlayers on the CO_2_R^28^. A rational choice of such an electrode is not easy and requires a clear consensus within the community. This also involves efficient discussions with industrial manufactures to ensure batch-to-batch reproducibility and quality control among the generated benchmark electrodes.

## Component-scalability

Scalability of the respective components must likewise be thought of. A catalytic/co-catalytic material that is only available in microgram scale in the required morphology or purity has minimal utility for application. Moreover, as electrocatalysts are often true nanomaterials, the production and handling of highly functional particles <1 µm, including colloidal process engineering, coating and drying is a research challenge that must be tackled simultaneously. We recommend that scalability to at least the kilogram scale should be considered right from the beginning, including the early involvement of process engineering and production technologies. The same issues of reproducible scaling are encountered for GDE fabrication, since thousands of m² electrodes can only be realized together with chemical and mechanical engineering, e.g., developing continuous and automated spray coating and roll-to-roll processes.

## Carbon balance

We briefly touched upon the issue of carbon balance above, measuring CO_2_ crossover via an internal standard and analysis of the anode gas-matrix. Nevertheless, it is currently unclear where the actual carbon balance lies in terms of scaled systems. Taking ethylene as an example, theoretically for 1 t of electrochemically produced ethylene, 3 t to 6 t of CO_2_ could be mitigated depending on the employed electrolyzer cell. Nevertheless, recent results suggest that CO_2_ electrolysis can compete in the future only by significant improvements in the current density and energy efficiency at which electrolyzers are operating^29,30^. Simultaneously, valorization of the anode stream is necessary to further increase the net CO_2_-negative by means of electricity savings. However, in contrast to water oxidation, electro-organic oxidations are associated with location limitations. The employed substrates and targeted products must be economically deliverable to and away from the electrolyzer, respectively. Specifically, while some technoeconomic analyses have been performed, these focus mainly on the North-American market, underlying the need to develop analytic models based on topography for different regions of the world^31^. Such models would allow for more efficient collaborations between academia and industry, generating heat-maps of markets/locations with elevated CO_2_ emissions, access to cheap renewable electricity and in addition close proximity to bio-mass sources.

In this comment, we suggest pathways to bridge the gap between fundamental research and industrial demands to establish new disruptive technologies. We herein provide additional parameters that we realized are important from our collaborations with industrial partners. Also, we hope to inspire the community to come up with experimental protocols (as for e.g., batteries) and benchmark electrodes (e.g., as Pt/C for PEM electrolysis) to make academic developments relevant.
